# Waste Oyster Shell/Graphene Oxide Composite as a Dual-Functional Soil Conditioner and SRF: Impacts on Soil pH and Nutrient Availability

**DOI:** 10.3390/nano15211666

**Published:** 2025-11-01

**Authors:** Hsuhui Cheng, Yuxing Xian, Yetong Lu, Ziying Zhang, Yishi He, Xiangying Hao

**Affiliations:** 1 School of Environmental and Chemical Engineering, Zhaoqing University, Zhaoqing 526061, China; xyx2587404642@163.com (Y.X.); 18922181874@163.com (Y.L.); 18319555433@163.com (Z.Z.); heyishi624@gmail.com (Y.H.); 2 Guangdong Provincial Key Laboratory of Eco-Environmental Studies and Low-Carbon Agriculture in Peri-Urban Areas, College of Environmental and Chemical Engineering, Zhaoqing University, Zhaoqing 526061, China

**Keywords:** oyster shell powders, graphene oxide, soil conditioners, slow-release fertilizer

## Abstract

Graphene oxide (GO) was prepared by a waterless synthesis route to generate GO sheets, which were then applied to coat calcined oyster shell with fertilizer (OSF) pellets, resulting in the creation of an OSF-GO particle. The GO sheets (ID/IG = 0.86) were characterized by Raman spectroscopy, which showed that the GO-coated OSF pellet features a compact coating approximately 13.68 μm thick. SEM and AFM analyses revealed that the GO sheets displayed a monolayer configuration with a crinkled topography (about 0.91 nm). The EDS analysis confirmed that the core was primarily composed of Ca, K, P, O, N, and C elements. The hydroponic experiment results showed that a GO concentration of 80 mg/L significantly enhanced plant height, stem thickness, and root length in loose-leaf lettuce, while higher concentrations induced oxidative stress. In pot experiments, the OSF-GO composite effectively raised the soil pH from 5.38 to 6.41 and improved nutrient availability. OSF-GO composite functions effectively as both a soil conditioner and slow-release fertilizer (SRF), simultaneously remediating degraded soils and optimizing nutrient delivery.

## 1. Introduction

Soil acidification represents a significant form of soil degradation capable of inducing irreversible alterations to soil properties [1]. This is predominantly attributed to the leaching of base cations such as K^+^, Ca^2+^, Na^+^, and Mg^2+^ caused by heavy rainfall or flooding [2]. Over-application of nitrogenous fertilizers [3,4], and intensive agricultural activities also contribute to declining soil fertility and overall soil health [5]. Guangdong Province is situated in southeastern coastal China and falls within a subtropical monsoon climate zone, characterized by warm, rainy conditions and prolonged summers. Under this climate, the soils of Guangdong were predominantly red soil and lateritic red soil, generally classified as acidic, with an average pH value of 5.41 [6]. The Guangdong Province Cultivated Land Fertilizer Station survey revealed a decline in soil quality across farmland, which was attributed to the overuse of land and fertilizer, loss of organic matter, and soil hardening [7]. In addition, with the development of the economy, the population of Guangdong has increased dramatically, and non-agricultural infrastructure projects have consumed extensive arable land, resulting in a significant reduction in arable land resources; problems such as contradictions in the food structure are becoming increasingly prominent [8]. Consequently, improving soil fertility, preventing soil degradation, optimizing nutrient cycling, and increasing nutrient utilization are crucial for sustainable agricultural development.

Oysters constitute a major economic source for the aquaculture industry on the southern coast of China [9]. In 2020, more than 5.82 million tons of oysters were produced in China, of which the total yield in Guangdong Province was 1.1 million tons, accounting for 17.1% of China’s total production, and the cultivation area reached 281.45 km^2^ (China Fisheries Statistical Yearbook 2023), generating a substantial quantity of waste oyster shells. Owing to their low recycling rate and limited economic value, the majority of these shell wastes, particularly oyster shells, were improperly disposed of, leading to significant environmental impacts and accelerating the degradation of coastal ecosystems [10,11].

Many studies have shown that oyster shells play an important role in remediating acidic soils. Wang et al. applied that a calcined oyster shell (COS) soil conditioner mitigates soil acidification in Shixia longan and chive cultivation, enhances soil fertility, and improves the yield and quality of fruits and vegetables [12]. Li et al. utilized calcined oyster shell powder (COSP) mixed with acidic red clay, and the results indicated that COSP could neutralize soil acidity, strengthen soil consolidation, enhance soil organic matter content, and enhance the flavor and quality of Guanxi pomelo [13]. The primary component of oyster shell powder (OSP) is calcium carbonate (CaCO_3_) [14], which is slightly soluble in water and alkaline [15], mitigates acidification induced by over-fertilization, and restores exchangeable Ca and Mg levels in soils [16]. However, relying solely on lime conditioners over the long term can lead to soil crusting and nutrient disorders [16,17]. Developing ecologically friendly soil remediation technology and timely nutrient supplementation technology for the soil is crucial for soil quality and agricultural sustainability.

Nanotechnology has emerged as a viable strategy for improving the nutrient use efficiency of agricultural fertilizers while concurrently mitigating environmental challenges and addressing the limitations associated with conventional fertilizers [18,19]. As a novel nanomaterial, graphene has found extensive applications across various fields owing to its advantageous physicochemical properties. Graphene oxide (GO) is a graphene derivative owing to its abundant oxygen-bearing hydrophilic functional groups [20,21], including carboxyl, carbonyl, epoxy, and hydroxyl groups, which are amenable to modification [22,23]. Many studies have shown that GO possesses a very high surface area and charge density, enabling it to bind to a large number of nutrient ions required by plants [24]. Yang et al. demonstrated that the utilization of GO could enhance chlorophyll content and relative water content (RWC), thus promoting improved plant growth potential and yield [25]. Lalwani et al. reported that lignin peroxidase, a ligninolytic enzyme secreted by white-rot fungi, is capable of efficiently degrading GO in soil, transforming it into non-toxic components, and consequently preventing secondary environmental contamination [26]. Safikhan et al. further indicated that the use of GO as an agricultural amendment can significantly enhance the physicochemical quality of the soil [27]. In our previous study [28], we successfully developed a slow-release fertilizer (SRF) by encapsulating NPK compound pellets with GO sheets using a waterless synthesis technique. This method provides plants with essential nutrients while also reducing environmental pollution.

Currently, several studies have demonstrated that OSP is an effective soil conditioner, while GO can function as an SRF due to its tunable nutrient loading and release properties. However, no reports have combined OSP with GO as a dual-purpose material for soil conditioning and SRF to enhance soil quality. Consequently, this study aimed to assess the efficacy of OSF-GO composites using loose-leaf lettuce as a model crop. This research not only promotes the reuse of waste oyster shells from aquaculture but also addresses environmental pollution and land disposal challenges faced by coastal communities.

## 2. Materials and Methods

### 2.1. Preparation of GO Sheet

In this study, a large-area GO sheet was prepared using a raw material derived from fine flake graphite (600 mesh particle size (≥90%), 99% carbon basis) obtained from Gongyi Baichuan Environmental Protection Engineering Co., Ltd. (Gongyi, China), and oven-dried at 80 °C for 24 h before being used. The GO material was synthesized using a waterless synthesis method, involving multiple wash cycles using deionized water and ethanol until the supernatant reached neutrality, thereby preventing minimizing residual ion accumulation. Subsequently, the resultant GO solution was freeze-dried for 48 h to yield the GO sheet material. More details on preparing the GO sheet material are described in our previous study [28].

### 2.2. Preparation of OSF-GO Particle

**Figure 1:**
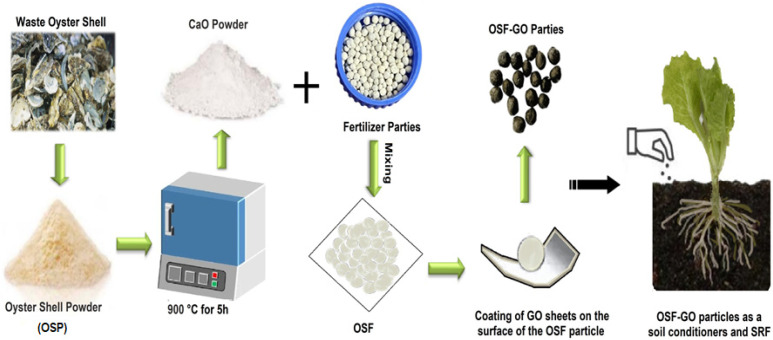
Schematic illustration of preparation of OSF-GO particle as a soil conditioners and SRF.

Waste oyster shells (WOS) were obtained from a local restaurant in Zhaoqing, Guangdong Province, China. The collected WOS was initially washed with standard tap water and subsequently with deionized water (SBK-RO-A04, Chaojing Water Treatment Technology Co., Ltd., Dongguan, China) to remove extraneous substances and impurities. Following the washing process, the WOS was desiccated in an electric heating constant-temperature oven (WT-D2, Lichen Technology Co., Ltd., Shaoxing, China) at 120 °C for 8 h. Upon complete desiccation, oyster shells were ground in a high-speed mill and sieved to a particle size of less than 100 μm (average size: 70 μm). The resultant powder was placed in a muffle furnace (LC-RF1-12TP; Lichen Technology Co., Ltd., Shaoxing, China) and heated to 900 °C to convert CaCO_3_ into CaO. Subsequently, the fine powder was calcined for 5 h and allowed to cool to ambient temperature to obtain OSP. Predetermined quantities of OSP and general-purpose NPK compound fertilizer (GF) were then combined in various proportions. Next, 2 mL of deionized water was gradually added to each OSP/GF mixture at room temperature, while stirring with a magnetic stirrer for 5 min until gelatinization occurred. The mixture was subsequently dried overnight in a constant-temperature oven maintained at 50 °C. After drying, the OSP/GF composite was homogenized with a mortar and pestle and compressed into a 60 mg pellet form, referred to as the OSF particle, using a desktop pelletizer (YK 60, Tianhe Machinery Equipment Co., Ltd., Shanghai, China). Following this, the OSF pellet was carefully handled using fine-tip forceps, and mechanical strength was applied to coat its surface with GO sheets, resulting in the creation of a GO-coated OSF pellet, referred to as the OSF-GO particle, as shown in Figure 1.

### 2.3. Characterization

The chemical composition and nanostructure of GO were measured using the HORIBA JY LabRAM HR Evolution Raman spectrometer (Horiba, Kyoto, Japan), which has a laser wavelength of 532 nm and covers the spectral range from 1000 to 3500 nm cm^−1^. GO morphology was characterized using atomic force microscopy (AFM) (Dimension Edge, Bruker, Billerica, MA, USA; with tapping mode using an OTESPA-R3 silicon probe, Bruker). A scanning electron microscope (SEM, Zeiss Sigma 300 FE-SEM, Oberkochen, Germany) was operated at an accelerating voltage of 30 kV to examine the particle morphology and size. Energy Dispersive X-ray Spectroscopy (EDS, Bruker Quantax XFlash SDD 6/30) at 15 KV was employed to identify the chemical elements present in the OSF-GO composite.

### 2.4. Seedling Exposure

The seeds of the loose-leaf lettuce seedlings used in this study were obtained from Dadi Agriculture Co., Ltd., based in Meizhou, Guangdong Province, China. Loose-leaf lettuce seedlings (8 days old) that exhibited uniform growth were selected for the experiments. The seedlings were cultivated in 360 mL disposable plastic cups and placed in an outdoor growth chamber. The environment was kept in a 16 h light and 8 h dark cycle, featuring daytime and nighttime temperatures of 25 °C and 18 °C, respectively, and a relative humidity of 60%. Then, a solution containing GO was placed in a plastic cup, and the seedlings were exposed to five distinct GO concentrations: 0, 20, 50, 80, and 110 mg/L. Following a 30 day exposure period, the roots and shoots were carefully separated. The samples were then rinsed with deionized water to remove residual growth medium and gently blotted with absorbent paper to eliminate surface moisture. Root and shoot lengths, along with fresh and dry biomass, were subsequently measured and recorded. Finally, the response of seedling growth was chosen as the optimal concentration of GO for further pot culture experiments.

### 2.5. Pot Experiments

Soil samples were obtained from an agricultural field located in Zhaoqing, Guangdong Province, China (111°77′ E, 23°14′ N), at a depth of 0~20 cm. According to soil classification, the site represents a typical acidic lateritic soil. Following air-drying, the samples were subsequently oven-dried, pulverized, and sieved through a 2.0 mm mesh to eliminate root fragments and gravel. The processed soil was then analyzed for alkali-hydrolyzable nitrogen (N), available phosphorus (P), available potassium (K), and pH levels.

Soil pH was determined by mixing soil samples with deionized water at a 1:1 ratio, followed by thorough stirring and measurement using a pH meter (PHS-25, INESA, Shanghai, China). Alkali-hydrolyzable N in soil was quantified with an automated Kjeldahl analyzer (Model K1160, Hanon Instruments Co., Ltd., Jinan, China) in accordance with the Chinese regional standard DB64/T 1734–2020 [29]. Briefly, 3 g of air-dried soil, sieved through a 2 mm mesh, was placed in a 750 mL digestion tube; the instrument automatically added 40 mL of NaOH solution (400 g/L) and 30 mL of boric acid-indicator solution, followed by steam distillation and automated titration with 0.02 mol/L HCl. Available P in soil was extracted using 0.5 mol/L NaHCO_3_ and quantified via the molybdenum blue method (NY/T 1121.7–2014) [30] with a UV–Vis spectrophotometer (Model Genesys 180, Thermo Fisher Scientific, Waltham, MA, USA). Available K in soil was extracted using 1 mol/L ammonium acetate and determined via flame photometry (NY/T 889–2004) [31] with a flame atomic absorption spectrophotometer (Model AA-7000, Shimadzu, Kyoto, Japan).

Loose-leaf lettuce seedlings were obtained from Guangdong Dadi Agriculture Co., Ltd. (Guangzhou, China). Select lettuce seedlings (20 days old) with similar plant height and healthy growth, and plant them in foam boxes (size: length × width × height 46 cm × 34 cm × 28 cm). Each box had 30 kg of air-dried lateritic soil. The experiment consisted of three treatments, applied under a completely randomized design with four replicates for each treatment across 12 boxes. The OSF-GO particles were prepared by combining GF with calcined OSP, which is predominantly composed of CaO. The GF:OSP mass ratios were 2:1 for OSF-GO1 (40 mg GF + 20 mg OSP) and 1:1 for OSF-GO2 (30 mg GF + 30 mg OSP), as detailed in Table 1. Each 60 mg OSF pellet was surface-coated with GO at a concentration of 2.5 mg/L, corresponding to approximately 0.15 mg GO per pellet.

### 2.6. Statistical Analysis of Data

The study employed a completely randomized design. Data were analyzed using one-way ANOVA with GraphPad Prism 10 software, followed by Tukey’s HSD test for post hoc comparison to identify significant differences among treatment means. A significance level of *p* < 0.05 was adopted.

## 3. Results and Discussion

### 3.1. Morphology and Structure of GO Sheet

**Figure 2:**
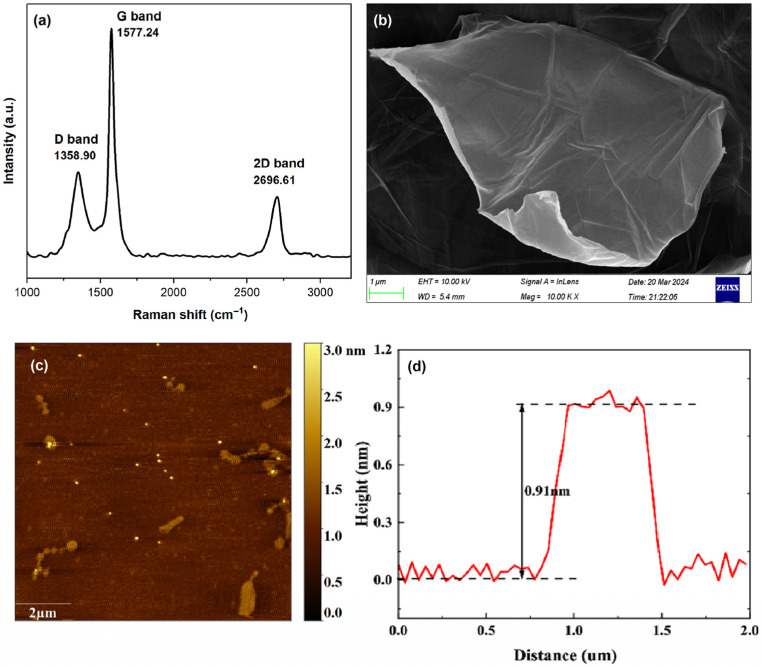
(**a**) Raman spectra of GO sheet, (**b**) SEM images of GO sheet, (**c**) AFM images of GO sheets topography, and (**d**) height profile.

Raman spectroscopy is an extensively utilized method for characterizing carbon products, primarily because conjugated carbon-carbon double bonds (C=C) lead to high Raman intensities [32]. The Raman spectra, as shown in Figure 2a, reveal a G band at 1577.24 cm^−1^; this band corresponds to the in-plane vibrational modes of sp^2^ hybridized carbon atoms and is indicative of a highly ordered sp^2^ carbon structure [33,34]. In addition to the G band, a prominent D band appears at 1358.90 cm^−1^, which is associated with the sp3 hybrid structure and signifies the presence of structural defects at the edges of graphene and disordered carbon [35,36]. It is well established that the size of the defect-free sp^2^ cluster regions is inversely related to the ratio of the integrated intensities of the D and G bands (ID/IG) [37]. For high-quality samples, the ID/IG intensity ratio is typically less than 2 [38]. In the present study, the waterless synthesized GO sheet has an ID/IG intensity ratio of 0.86. These results indicate similarities between the ID/IG intensity ratios reported by Chen et al. [36]. The 2D band is another important parameter for assessing the number of layers in GO samples. In the present study, the peak of the 2D band at 2696.61 cm^−1^ confirmed that the synthesized GO consists of a single-layer structure.

The surface morphology of the synthesized GO sheets was examined by scanning electron microscopy (SEM) [39]. As illustrated in Figure 2b, the waterless synthesized GO sheet sample exhibited a layered structure with highly sharp edges that are folded onto themselves, resulting in a wrinkled surface.

To conduct a more detailed investigation of the sizes and thicknesses of GO sheets, atomic force microscopy (AFM) was conducted in tapping mode. This mode was employed to prevent the dislocation of GO sheets. Figure 2c,d presents the AFM topography and the corresponding height profile of the GO sheets. Samples were prepared from the dispersion of GO in ethanol and then dried on mica substrate. As can be seen, GO sheets similar to SEM images have highly sharp edges. AFM characterization of the dispersion state of GO sheets revealed their uniform distribution on the mica substrate, with lateral dimensions extending into the micrometer scale. The height profiles represented by the straight line in Figure 2d indicate that single-layer GO on the mica surface is approximately 0.91 nm, which is aligned with the typical thickness of less than 1 nm, as reported in the existing literature [37,40].

### 3.2. SEM-EDS Analysis of OSF-GO Particle

**Figure 3:**
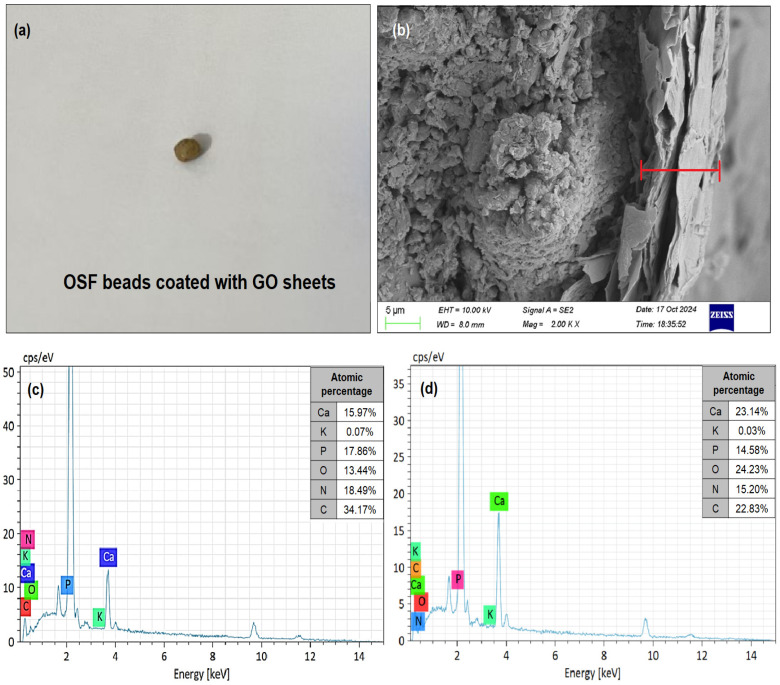
(**a**) OSF beads coated with GO sheets, (**b**) cross-sectional SEM image of the OSF-GO particle, (**c**) EDS spectra of OSF-GO1, and (**d**) OSF-GO2.

Figure 3a,b show the appearance of the OSF beads coated with GO sheets and SEM images of the cross-sectional GO-coated OSF particles, respectively. As expected, high-magnification SEM revealed the surface morphology of GO sheets coated with OSF particles, revealing a dense GO film. Examination of the cross-sections of GO sheets coated with OSF particles revealed a film thickness of approximately 13.68 μm (Figure 3b). EDS analysis confirmed that the two different constituents of the OSP material ratio (see Table 1) for the OSF-GO1 and OSF-GO2 particles were essentially Ca, K, P, O, N, and C elements. The results show that the elemental weight percentage for the synthesized OSF-GO1 and OSF-GO2 particles composition is as follows: 15.97 and 23.14 wt % of Ca, 0.07 and 0.03 wt % of K, 17.86 and 14.58 wt % of P, 13.44 and 24.23 wt % of O, 18.49 and 15.20 wt % of N, and 34.17 and 22.83 wt % of C, respectively. Figure 3c,d illustrate the EDS analysis of the synthesized OSF-GO1 and OSF-GO2 particles, respectively.

### 3.3. Evaluation of Growth Parameters in Hydroponic Systems

#### 3.3.1. Effect of GO Concentration on Growth and Development of Loose-Leaf Lettuce Seedlings

**Figure 4:**
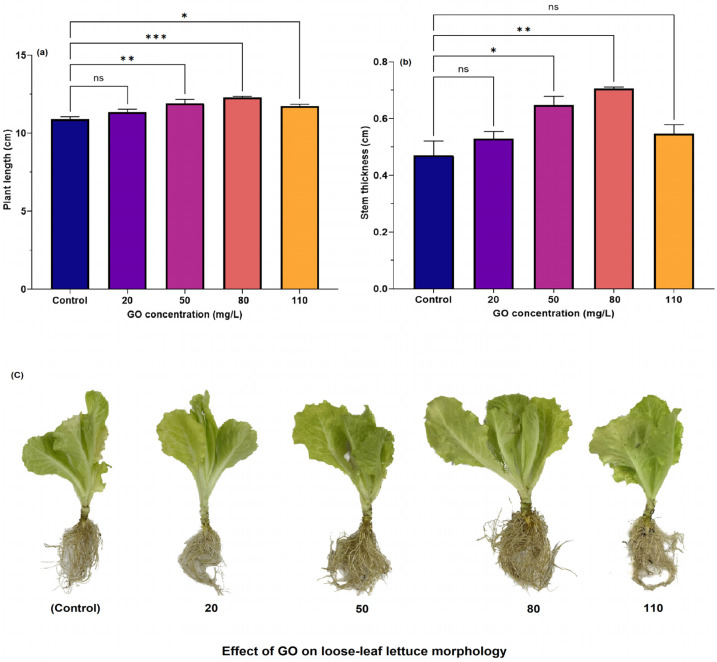
Impact of varying GO concentrations on (**a**) plant seedling length and (**b**) stem thickness of loose-leaf lettuce after 20 days of treatment. (**c**) Effect of GO on loose-leaf lettuce morphology. Statistical significance levels: *** *p* < 0.001; ** 0.001 < *p* < 0.01; * 0.01 < *p* ≤ 0.05. n.s. denotes non-significance where *p* > 0.05.

This section aims to identify the optimal concentration of GO that minimizes adverse effects on root growth in loose-leaf lettuce seedlings, thereby providing a foundation for subsequent pot experiments. To this end, seedlings were treated with GO at concentrations ranging from 0 mg/L (control group) to 110 mg/L, facilitating a systematic examination of the impact of varying GO concentrations on plant growth and development. Figure 4a demonstrates that the plant length of loose-leaf lettuce seedlings exhibited a significant increase with increasing GO concentrations extending to 80 mg/L. Relative to the control, plant length was significantly greater at 50 mg/L (*p* < 0.01) and 80 mg/L (*p* < 0.001), with the average plant length observed at 80 mg/L. However, at 110 mg/L, the plant length showed a slight decline, with no significant difference compared to the 80 mg/L group, though it was still markedly higher than the control group (*p* < 0.05). The results demonstrate that low GO dosages posed no threat to plant health [41], emphasizing the role of the nanomaterial at a suitable dosage for enhancing plant growth.

Similarly, our study revealed that the control group exhibited an average stem thickness of 0.47 cm, which increased to 0.53 cm (12.8%, *p* > 0.05) at a concentration of 20 mg/L GO, and further to 0.65 cm (38.3%, *p* < 0.05) at 50 mg/L GO. The maximum stem thickness of 0.71 cm (51.1%, *p* < 0.01) was recorded at 80 mg/L GO. These results demonstrated a positive correlation between stem thickness and leaf morphological characteristics, suggesting that low GO concentrations enhance stem and leaf biomass [41], potentially increasing stem thickness and thereby improving overall plant structural integrity and stability (see Figure 4c for morphological changes in GO exposed loose-leaf lettuce). However, at 110 mg/L GO, stem thickness declined to 0.55 cm (17.0%, *p* > 0.05), indicating that high GO concentrations may restrict plant growth due to toxic influences or oxidative stress, which aligns with existing literature [25,42].

#### 3.3.2. Effect of GO Concentration on Root Growth

**Figure 5:**
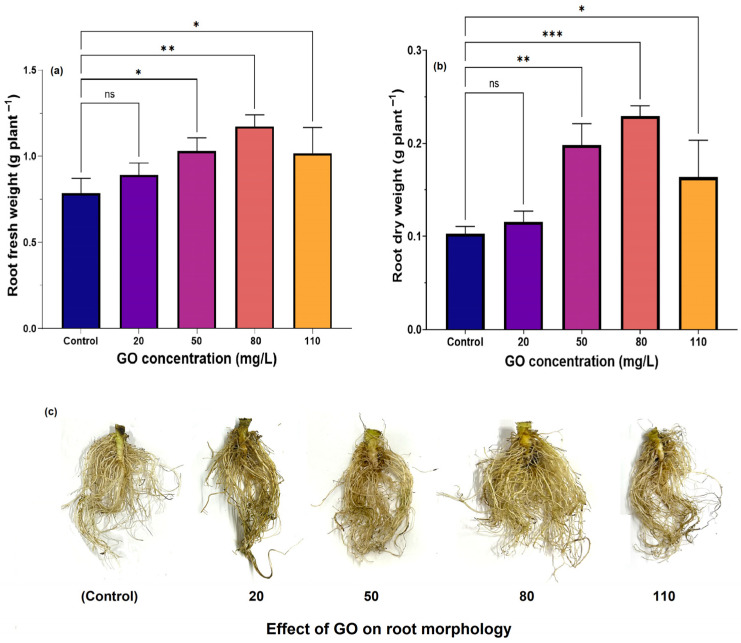
Impact of varying GO concentrations on (**a**) root fresh weight and (**b**) root dry weight of loose-leaf lettuce after 20 days of treatment. (**c**) Effect of GO on root morphology. Statistical significance levels: *** *p* < 0.001; ** 0.001 < *p* < 0.01; * 0.01 < *p* ≤ 0.05. n.s. denotes non-significance where *p* > 0.05.

Root elongation plays a vital role in plant growth and development. As illustrated in Figure 5a, the average root fresh weight significantly increased from 0.784 g/plant in the control group to 1.029 g/plant at 50 mg/L GO (*p* < 0.05), reaching 1.172 g/plant at 80 mg/L GO (*p* < 0.01). Contrary to this increasing trend, a reversal occurred at 110 mg/L, where the root fresh weight significantly decreased to 1.017 g/plant (*p* < 0.05). A similar pattern was observed for root dry weight (Figure 5b) and the number of root tips (Figure 5c), with the highest values recorded at 80 mg/L GO, the optimal GO concentration for root development, followed by a decline at 110 mg/L. These results indicate that, similar to typical growth regulators, GO exhibits a concentration-dependent effect on plant growth, and thus, an optimal concentration exists to elicit this response [25].

In contrast, higher GO dosages harmed root structure and impeded nutrient absorption in the roots, ultimately suppressing root development [43]. Shen et al. similarly reported that GO exerted a significant impact on rice root growth, with high concentrations of GO applied to five rice varieties resulting in reduced development of root length, fresh weight, and dry weight [44]. This observation suggests that elevated GO concentrations, particularly at higher doses, could prompt the formation of reactive oxygen species (ROS) within plant structures, thus causing harm to cells [45]. Therefore, subsequent pot experiments were conducted with a GO concentration set to 80 mg/L to avoid triggering oxidative stress responses.

### 3.4. Functional Test of OSF-GO Particles in Pot Experiments

The level of soil acidity or alkalinity, as measured by pH, serves as a key attribute that substantially affects nutrient availability and plant growth [46]. Prior to the pot experiment, soil samples were collected from the study site in January 2024, with measured pH values ranging from 5.31 to 5.38. According to China’s soil environmental quality standard [47], the soil was classified as strongly acidic (pH < 5.5). From Table 2, it is observed that, after 80 days of treatment, the soil pH value increased from 5.38 in the control group CF to 6.41 and 6.30 in the OSF-GO1 and OSF-GO2 treatments, respectively. The results indicate that OSF-GO composite material, when doped with calcined OSP, serves as an effective soil amendment for pH elevation and mitigating soil acidification, which is significantly effective in restoring pH levels in acidic soils [13]. The pH level of soil governs the quantity of nutrients and chemicals dissolved that are soluble in soil water [48], with each nutrient achieving optimal availability when soil pH ranges from 5.5 to 7.5 [49], thereby facilitating greater nutrient availability for lettuce growth.

In terms of plant morphology, this study showed that OSF-GO1 and OSF-GO2 treatment samples significantly increased plant height (28.9 ± 1.91 cm and 26.1 ± 2.23, respectively), root length (9.2 ± 0.88 cm and 8.1 ± 1.07, respectively), and stem thickness (2.8 ± 0.17 cm and 2.1 ± 0.33, respectively) when compared to the control group CF sample. Table 2 shows that the employment of GO effectively promotes plant growth, likely by enhancing root system development and structural stability. Moreover, when GO was combined with OSP to form the OSF-GO composite material, markedly elevated soil pH was achieved, thereby optimizing the bioavailability of key nutrients within their optimal pH ranges. The results showed that OSF-GO composite nanomaterials can produce a synergistic effect on plant growth as a soil conditioner and SRF. Although the effect observed for OSF-GO2 is slightly weaker than that for OSF-GO1, the results still show a positive trend.

In addition to soil pH, the availability of essential macronutrients such as N, P, and K is critical for sustaining microbial metabolic activity and supporting plant respiratory functions. As shown in Table 2, the OSF-GO1 and OSF-GO2 treatments significantly elevated the initial soil pH from 5.38 to 6.41 and 6.30, respectively. Over the 80-day incubation period, residual alkali-hydrolyzable N decreased by 12.8% and 15.4% compared to the CF treatment, likely due to pH adjustment into the optimal range for nitrification (5.5–7.5) [50], thereby promoting the conversion of mineral N into nitrate (NO_3_^−^). Since NO_3_^−^ carries a negative charge, it is less likely to be adsorbed by soil colloids [51], enhancing its availability to plants and contributing to a notable increase in plant height.

Moreover, P ranks as the second most critical nutrient for plants, following N [52]. Acidic soils can limit the uptake of essential nutrients by plants [53], particularly P, which tends to bind to Al^3+^ and Fe^3+^ oxide structures, thereby significantly suppressing root development [54]. In this study, OSF-GO1 and OSF-GO2 treatments resulted in a 13.7% (159.9 mg/kg) and 17.9% (152.3 mg/kg) reduction in residual available P, respectively, compared to the CF treatment. Correspondingly, root lengths significantly increased to 9.2 cm and 8.1 cm under OSF-GO1 and OSF-GO2, respectively, whereas the CF group exhibited an average root length of only 6.3 cm. These findings further support the notion that the OSF-GO composite effectively alleviates soil acidity and enhances the bioavailability of dihydrogen phosphate (H_2_PO_4_^−^/HPO_4_^2−^), thereby facilitating its uptake by plants.

K is an essential macronutrient involved in various physiological processes [55], including root elongation, osmotic regulation, stomatal function, and phloem transport of photoassimilates [56,57]. K deficiency exacerbates N metabolic imbalances, resulting in suppressed photosynthetic efficiency, impaired nutrient mobility, and lower yields [58]. In this study, the OSF-GO1 group exhibited the lowest residual soil K content (91.1 ± 5.95 mg/kg). It showed a 46% increase in root length compared to the chemical fertilizer (CF) group. This suggests that OSF-GO1 not only enhanced the utilization efficiency of available K but also facilitated its translocation within the plant system. This effect may be linked to the activation of plasma membrane H^+^-ATPases [59], which subsequently lowers the pH of the cell wall. Li et al.’s study indicated that acidification is known to promote cell wall loosening, thereby enhancing root elongation and increasing nutrient absorption capacity [60]. The role of this mechanism is further supported by the observed increase in root length following OSF-GO1 treatment. In addition, OSF-GO1 significantly improved shoot growth parameters. Specifically, stem thickness (2.8 ± 0.17 cm) and plant height (28.9 ± 1.91 cm) were markedly higher than those in the CF group. These enhancements are consistent with the known roles of K in regulating stomatal conductance and optimizing the phloem loading of carbohydrates, both of which contribute to efficient carbon assimilation and biomass production [61]. Furthermore, the increased soil pH (6.41) in the OSF-GO1 treated soil may have also enhanced the availability of K from clay minerals into the soil solution [62], thereby improving the fraction of K available for plants.

**Figure 6:**
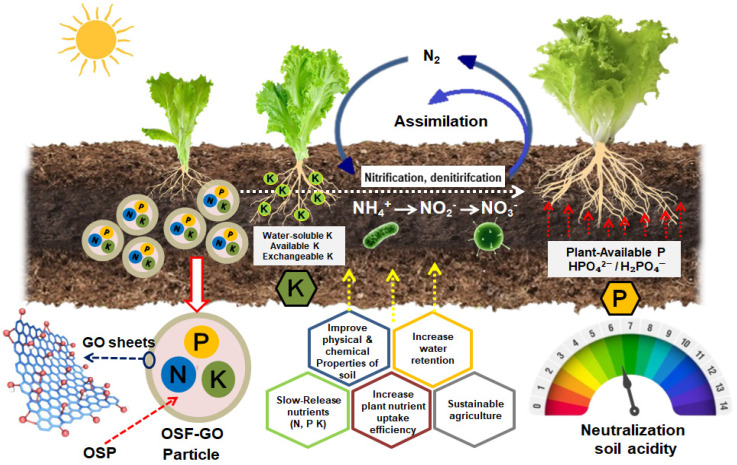
Schematic of the OSF-GO composite action mechanism as dual-functional soil conditioner and SRF.

In conclusion, this study demonstrates a synergistic mechanism in the OSF-GO composite that couples soil conditioning with nutrient delivery (see Figure 6). The OSP component, derived from waste oyster shells, acts as a natural alkaline amendment, neutralizing soil acidity and raising the pH from 5.38 to approximately 6.41, thereby creating a supportive environment for plant growth in the rhizosphere. Meanwhile, the GO layer, prepared via a waterless synthesis route, exploits its extensive surface area and oxygen-bearing functional groups to facilitate controlled release of primary macronutrients N, P, and K. This dual-function design not only enhances pH buffering and nutrient bioavailability but also aligns with principles of waste valorization and green nanomaterial synthesis. These improvements positively correlate with significant gains (*p* < 0.05) in plant height, stem diameter, and root length (as shown in Table 2), highlighting the potential of the OSF-GO composite as an environmentally conscious approach for sustainable soil agriculture technology.

## 4. Conclusions

In the present research, GO was prepared by waterless synthesis, yielding high-quality GO sheets as confirmed by Raman spectroscopy (ID/IG = 0.86), SEM, and AFM, with a monolayer thickness of approximately 0.91 nm. Compared with the traditional Hummers method, this waterless synthesis is more environmentally friendly, operationally simpler, lower in cost, and avoids the use of hazardous reagents. Furthermore, OSP derived from aquaculture waste was valorized as a low-cost, calcium-rich support for the fertilizer matrix, aligning with circular economy principles. Hydroponic experiments showed that a GO concentration of 80 mg/L significantly enhanced plant height, stem thickness, and root length in loose-leaf lettuce, whereas 110 mg/L induced growth suppression, likely due to reactive oxygen species generation. Pot experiments demonstrated that the OSF-GO composite effectively raised soil pH from 5.38 to 6.41 and improved nutrient availability through controlled release, thereby promoting plant growth while mitigating the environmental drawbacks associated with conventional fertilizers. This work underscores the potential of integrating green synthesis with waste valorization for sustainable agricultural applications.

## Figures and Tables

**Table 1:** Constitute of the OSF-GO particles.

Treatment Samples	Particle Constitute			
	GF (mg)	CaO (mg) (from Calcined OSP)	GO as a Coating on Metals (mg/L)	GF:OSF Ratio
GF	60	0	0	--
OSF-GO1	40	20	2.5	2:1
OSF-GO2	30	30	2.5	1:1

Note: CaO was obtained by calcining OSP at 900 °C.

**Table 2:** Effects of OSF-GO treatment on soil pH and nutrient availability in loose-leaf lettuce.

Treatment Samples	Plant Morphology	Plant Height	Stem Thickness	Root Length	pH	Alkli Hydrolysable N	Available P	Available K
		cm				mg/kg		
CF	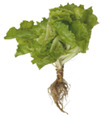	14.3 ± 0.60	1.4 ± 0.08	6.3 ± 0.43	5.38 ± 0.07	229.7 ± 6.18	185.4 ± 7.22	117.8 ± 4.62
OSF-GO1	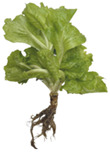	28.9 ± 1.91	2.8 ± 0.17	9.2 ± 0.88	6.41 ± 0.11	200.4 ± 8.36	159.9 ± 6.35	91.1 ± 5.95
OSF-GO2	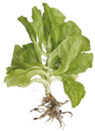	26.1 ± 2.23	2.1 ± 0.33	8.1 ± 1.07	6.30 ± 0.06	194.4 ± 4.68	152.3 ± 4.79	99.9 ± 4.70

## Data Availability

The original data presented in this work are contained in the article, and further questions can be directed to the corresponding author.
